# Measured Glomerular Filtration Rate: The Query for a Workable Golden Standard Technique

**DOI:** 10.3390/jpm11100949

**Published:** 2021-09-24

**Authors:** Marijn M. Speeckaert, Jesse Seegmiller, Griet Glorieux, Norbert Lameire, Wim Van Biesen, Raymond Vanholder, Joris R. Delanghe

**Affiliations:** 1 Department of Nephrology, Ghent University Hospital, 9000 Ghent, Belgium; griet.glorieux@ugent.be (G.G.); norbert.lameire@ugent.be (N.L.); wim.vanbiesen@ugent.be (W.V.B.); raymond.vanholder@ugent.be (R.V.); 2 Research Foundation Flanders, 1000 Brussels, Belgium; 3 Department of Laboratory Medicine and Pathology, University of Minnesota, Minneapolis, MN 55455, USA; jseegmil@umn.edu; 4 Department of Diagnostic Sciences, Ghent University, 9000 Ghent, Belgium; joris.delanghe@ugent.be

**Keywords:** contrast media, glomerular filtration rate, inulin, radioactive tracers

## Abstract

Inulin clearance has, for a long time, been considered as the reference method to determine measured glomerular filtration rates (mGFRs). However, given the known limitations of the standard marker, serum creatinine, and of inulin itself, and the frequent need for accurate GFR estimations, several other non-radioactive (iohexol and iothalamate) and radioactive (^51^Cr-EDTA, ^99m^Tc-DTPA, ^125^I iothalamate) exogenous mGFR filtration markers are nowadays considered the most accurate options to evaluate GFR. The availability of ^51^Cr-EDTA is limited, and all methods using radioactive tracers necessitate specific safety precautions. Serum- or plasma-based certified reference materials for iohexol and iothalamate and evidence-based protocols to accurately and robustly measure GFR (plasma vs. urinary clearance, single-sample vs. multiple-sample strategy, effect of sampling time delay) are lacking. This leads to substantial variation in reported mGFR results across studies and questions the scientific reliability of the alternative mGFR methods as the gold standard to evaluate kidney function. On top of the scientific discussion, regulatory issues are further narrowing the clinical use of mGFR methods. Therefore, this review is a call for standardization of mGFR in terms of three aspects: the marker, the analytical method to assess concentrations of that marker, and the procedure to determine GFR in practice. Moreover, there is also a need for an endogenous filtration marker or a panel of filtration markers from a single blood draw that would allow estimation of GFR as accurately as mGFR, and without the need for application of anthropometric, clinical, and demographic characteristics.

## 1. Introduction

**Figure 1:**
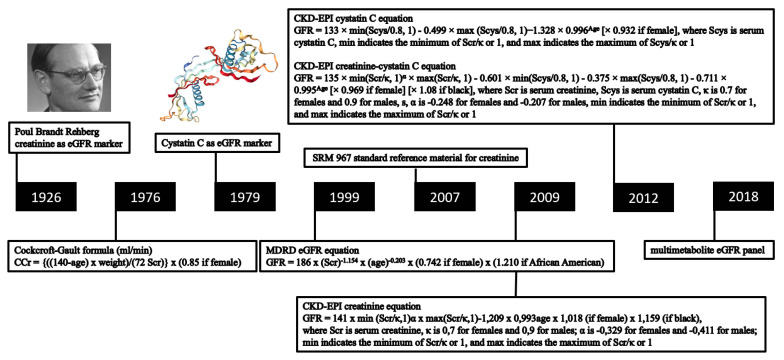
Overview of key moments in the development of GFR equations with endogenous filtration markers.

Accurate determination of the glomerular filtration rate (GFR) is essential for the diagnosis of early kidney disease. In clinical practice, GFR is typically estimated (eGFR) from blood concentrations of endogenous filtration markers (Figure 1) [1]. Despite all the diagnostic advances in GFR assessment, there remains a lack of accuracy for the current eGFR computational approaches, especially until GFR falls to <60 mL/min/1.73 m^2^ [2]. Plasma or urinary clearances of exogenous GFR markers are considered the most accurate way to evaluate kidney function (measured GFR, mGFR). This approach is typically desired in cases of substantially diverging anthropometric properties. Establishing mGFR is also required when accurate knowledge of kidney function is deemed essential, such as in patients receiving chemotherapy or in candidates for kidney donation [3,4,5,6]. As standardized protocols to perform this procedure are lacking, the mGFR value obtained by an improperly implemented protocol may be biased, which can lead to incorrect conclusions of donor suitability. Criteria for reliable exogenous GFR markers are the following: water-soluble, not bound to proteins, only excreted by the kidneys, 100% filtered through the glomerulus in subjects with a normal kidney function, and neither secreted nor absorbed by the kidney tubules. Several exogenous markers have been evaluated for this purpose including inulin, ^51^Cr-ethylenediamine tetraacetic acid (^51^Cr-EDTA), ^99m^Tc-diethylenetriamine pentaacetic acid (^99m^Tc-DTPA), ^125^I-iothalamate, and some non-isotopic “cold” markers (iothalamate and iohexol) [7,8]. This opinion paper provides an overview of these exogenous mGFR markers, along with their limitations, in chronic stable individuals. Given these hurdles, a multimetabolite eGFR panel derived from a single blood draw can probably estimate GFR at least as accurately as mGFR, without the need for specification of demographic and clinical characteristics, or a laborious procedure including repeated blood sampling and/or urine collection.

## 2. Exogenous Markers to Measure GFR

### 2.1. Inulin

**Figure 2:**
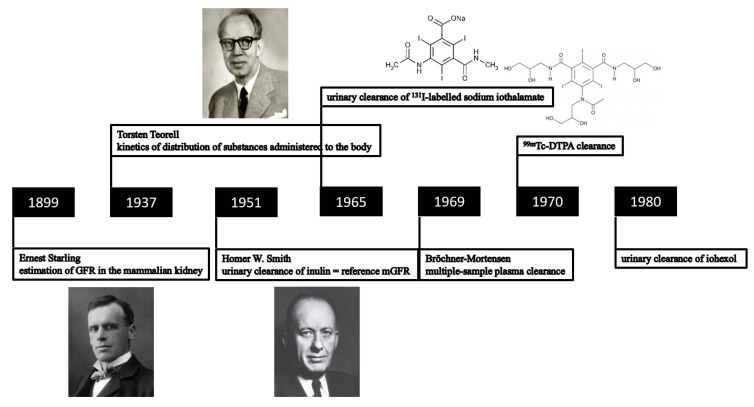
Timeline representing the evolution of the use of exogenous filtration markers for the determination of GFR.

For a long time, urinary inulin clearance over a 24 h period (Figure 2) has been considered as the reference method for GFR measurement [9]. Inulin clearance protocols should be conducted by trained personnel, and patients should present in the morning in a fasting state. After an intravenous inulin loading dose, a subsequent maintenance infusion is administered to achieve stable plasma concentrations (300–400 mg/L) during the entire clearance period. The collection of blood and urine samples should be carefully timed [10]. Oral water loading should be encouraged to increase urine flow, as large urinary volumes increase the accuracy of timed volumetric urine collections. When there is doubt whether the patient can void completely, bladder catheterization might even be required. This classical inulin clearance method is characterized by an analytical imprecision, with a coefficient of variation (CV) for repeated inulin concentration measurements of approximately 7%, as well as by a risk of protocol violations [11] as those are difficult to apply and invasive.

### 2.2. Non-Inulin Exogenous Markers

As inulin production and availability from European Union (EU) chicory crops is below market demand, and as the approach to determine inulin clearance is very laborious, most clinicians have abandoned this method and have replaced it with several alternative mGFR protocols, measuring the plasma and/or urinary clearance of radioactive (^51^Cr-EDTA, ^99m^Tc-DTPA, ^125^I iothalamate) or non-radioactive (iohexol (Omnipaque™, GE Healthcare Inc, Chicago, IL, USA, and Accupaque™, GE Healthcare Inc, Chicago, IL, USA) and iothalamate (Conray™, Mallinckrodt Inc, Staines, UK)) tracers. These alternative mGFR assays have reported CVs in the range of 5–15%, generally with higher values for urinary clearance than for plasma clearance methods based on the area under the curve (AUC) [12,13,14,15,16,17,18,19,20].

#### 2.2.1. Methodology and Procedure

##### Clearance Methodology

The methodology to measure GFR can influence the results of mGFR. The classical urinary clearance method is based on the administration of an exogenous marker by continuous infusion. Although urinary clearance remains the reference method, especially in patients with an abnormal extracellular volume (edema or ascites), this technique is cumbersome and impractical due to the difficulty to obtain accurate urine collections and mandatory bladder catheterization, especially in young children or older patients. For these reasons, there is often a preference to use the plasma clearance method, which has the best balance between physiology and feasibility and an acceptable concordance with urinary clearance [21]. Due to logistic reasons, plasma-based clearance assays with a one-compartment kinetic model are preferred over urinary clearance methods [22,23,24,25]. However, plasma disappearance protocols are influenced by the administered quantity of the marker, the timing of sample collection(s), and the length of the procedure and depend on proper calibration and assessment procedures [26,27].

##### The Timing Procedure of Plasma Sampling: Single- vs. Double- vs. Multiple-Sample mGFR

Different methodologies to measure GFR by plasma clearance have been published with a varying number and timing of plasma samples. In comparison with a continuous infusion, a single injection of an exogenous marker is more attractive from the practical point of view. Since the publication of the two cornerstone papers [28,29], describing the kinetics of the distribution of substances administered to the body, several researchers have performed a theoretical analysis of the kinetics of GFR markers by applying an open multicompartmental system after a single injection [30,31,32,33]. After the single injection, GFR can be calculated by multiple plasma sampling (eight or more) to determine the plasma clearance slope. A time–activity curve is plotted, dividing the dose by the AUC results in a calculated GFR. The timing is crucial in multiple-sample methods as plasma concentrations of exogenous markers will decrease according to two different exponential curves: the fast component corresponds to the distribution volume, whereas the slow component corresponds to the kidney clearance of the marker [34]. An accurate determination of GFR with this labor-intensive method is difficult and costly for routine clinical practice. Many simplified methods for clearance calculation have been proposed to reduce the number of plasma samples (single vs. double plasma sampling) while maintaining an acceptable degree of accuracy [35,36,37,38,39,40,41,42,43,44,45,46,47,48].

Several mathematical procedures have been proposed for mGFR calculation based on a single-sample collection method as an alternative for the multiple-sample procedure. Although the performance of the single-sample plasma clearance method is similar to the more complex multiple-sample plasma clearance, discrepancies are observed in some specific clinical situations: e.g., in patients with a low GFR (<30 mL/min) or with a very high BMI (≥40 kg/m²) [21]. GFR protocols should be customized according to the degree of kidney insufficiency and the volume status to optimize protocol accuracy [49]. Using the single-sample method, sample timing is fundamental and should be late (300–360 min after a single injection of iohexol if GFR is presumed to be 30–60 mL/min, and 600 or 1440 min after injection if GFR is <30 mL/min). By contrast, the sampling time should be early (180 min after the single injection) if GFR is presumed normal or supranormal. When the sample timing is adapted to the expected GFR, acceptable accordance (within 10%) has been reported between single-sample and multiple-sample procedures. However, the estimation of the extracellular volume is a predominant source of error with the single-sample method in patients with ascites or edema [34]. In the double-sample method, next to a delayed final blood collection at 8–24 h in patients with a low GFR, a delay in the early sample has also been recommended. This approach enhances the accuracy significantly compared to delaying only the final collection [24,50]. Several preferred iohexol methodologies have been proposed based on patient characteristics (Table 1), due to a delay in attaining the linear phase of the disappearance curve. The 2–4 h ^99m^Tc-DTPA protocol is associated with the least accurate GFR results and cannot be recommended [49].

##### Analytical Methods: Difference in Assays Used for GFR Marker Measurement

At this moment, there is a lack of standardization of the assays for measurement of plasma and urinary concentrations of exogenous GFR markers.

Analysis of the non-ionic contrast medium iohexol can be performed with several methods: liquid chromatography-tandem mass spectrometry (LC-MS/MS) [8], high-performance liquid chromatography-ultraviolet detection (HPLC-UV) [51,52], and X-ray fluorescence [8]. In an aqueous solution, iohexol exists as a mixture of isomers, which slowly interconvert and gradually equilibrate to an isomeric ratio [53]. A difference in the initial peak area ratios of endo (~13%) and exo (~87%) forms in aqueous solutions of freshly prepared iohexol powder calibrators is found in comparison to the endo (~26%) and exo (~74%) peak ratios after the equilibration period [27]. The duration and temperature of storage substantially impact isomer equilibration in stock solutions from iohexol powder, and this should be a crucial point of attention for laboratories preparing calibrators or controls from powdered crystalline iohexol. Heat sterilization, as used in commercially available iohexol solutions (such as Omnipaque™ and Accupaque™), might induce a rapid and stable equilibrium of the exo- and endo-iohexol ratio, which solves this problem by reaching the final equilibrium before the first use [54,55].

Procedure-dependent measurement differences have been reported when determining plasma iohexol clearance using LC-MS/MS vs. HPLC-UV. LC-MS/MS iohexol measurement resulted in a significantly lower plasma clearance in comparison with HPLC iohexol plasma clearance [56]. These differences in the reported plasma iohexol clearances might be explained by divergent registered concentrations by LC-MS/MS vs. HPLC-UV, related to a variable presence of endo- and exo-isomers of iohexol [27]. In the majority of HPLC-UV measurement procedures, separation and quantification are only based on the major (exo-iohexol) isomeric peak [26,51,57]. In contrast to HPLC-UV, LC-MS/MS measurement procedures typically do not separate iohexol isomers and quantify all forms of iohexol in one single integration event. This results in a constant total iohexol concentration measurement over time at every temperature or any isomer equilibration. In a study measuring only the exo-isomer, a minimal precision difference (<1%) between iohexol quantitation by HPLC-UV and LC-MS/MS was found, suggesting that both systems can generate near-identical results when performed with strict adherence to pre-defined procedures [27]. LC-MS/MS is theoretically a more sensitive and specific method but is more complex and costly in comparison with HPLC-UV [34].

Due to its lower sensitivity, the performance of X-ray fluorescence is inferior to HPLC-UV and is not recommended [58,59].

Plasma clearance methods rely on proper calibration and assessment procedures [26,27]. Calibration can be verified by blinded internal and external quality assessment (EQA) by blinded assessment of replicates of previously tested patient samples or by participation in an EQA/proficiency testing program. It should be mentioned that in the absence of serum or plasma matrix-based certified reference materials, inter-laboratory differences in iohexol concentrations of >10% have been reported, resulting in an equivalent 10% difference in mGFR in a direction opposite to that of the iohexol concentration measurement difference [26].

### 2.3. Radioactive Tracers

The plasma disappearance curve after a single, well-defined dose after a single injection of radioactive tracers such as ^51^Cr-EDTA, ^99m^Tc-DTPA, and ^125^I-iothalamate can be used to measure GFR [60]. The use of ^51^Cr-EDTA is associated with a lower workload for preparation and reference activities as the calibration reference curve is stable for several weeks with a long half-life (27.7 days), compared to only 6 h for ^99m^Tc-DTPA [61] and 3.2 h for ^125^I-iothalamate [62].

#### 2.3.1. Comparison between Clearance of Radioactive Tracers and Inulin Clearance

A high concordance with minor error has been observed between plasma ^51^Cr-EDTA-clearance and urinary as well as plasma inulin clearance [63,64,65,66,67,68,69]. A consistent underestimation in comparison to inulin clearance (5–15%) was observed with urinary ^51^Cr-EDTA clearance, suggesting tubular reabsorption [70]. There is moderately strong evidence for the accurate measurement of GFR by urinary and plasma clearance of ^51^Cr-EDTA [71].

Plasma inulin and ^99m^Tc-DTPA clearance curves were comparable [72], whereas others found that plasma inulin clearance exceeded that of ^99m^Tc-DTPA [73]. Plasma ^99m^Tc-DTPA clearance correlated well with urinary inulin clearance, but with an overestimation of 3.5 mL/min on average. Urinary ^99m^Tc-DTPA clearance was, on average, 3% lower than urinary inulin clearance [73]. Urinary ^125^I-iothalamate clearance showed a small positive bias in comparison with urinary inulin clearance, probably due to tubular secretion of iothalamate [74,75,76,77,78,79]. Other studies could not confirm this finding [80,81,82,83].

#### 2.3.2. Comparison between Clearances of Radioactive Tracers

Although small differences in mGFR results between radioactive tracers have been observed [61,84,85], they are clinically irrelevant in comparison with the higher intra-patient variability of GFR measurements.

#### 2.3.3. Limitations of the Use of Radioactive Tracers

Besides the fact that the use of radioactive tracers requires places for storage, administration, and disposal, several practical problems specific for each marker are observed. ^51^Cr-EDTA is not licensed for use as a filtration marker in some countries, such as the USA, which substantially limits the number of studies performed with this marker. Since 2018, a shortage of ^51^Cr-EDTA in Europe has driven a switch to ^99m^Tc-DTPA-based single-sample mGFR determinations. A major limitation for ^99m^Tc-DTPA mGFR protocols is the potential for dissociation of ^99m^Tc from DTPA, resulting in its binding to plasma proteins and leading to underestimations of GFR. The extent of dissociation is not predictable, leading to imprecision and bias. Besides its free filtration at the glomerulus with minimal tubular reabsorption, DTPA may undergo extrarenal elimination by the gut and liver. Moreover, no standardization of chelating kits and technetium generators exists, making mGFR comparisons among different institutions unreliable. As cold iodine is administered simultaneously with ^125^I-iothalamate to block thyroidal uptake, its use is precluded in people with known iodine allergies [3]. Finally, working with radioactive compounds is laborious, linked with many preventive safety measures, and therefore it is desirable and safer to avoid infusing radioactive compounds in patients.

### 2.4. Non-Ionic Monomeric Contrast Media

mGFR determination by examining the clearance of contrast media (iohexol or iothalamate) is a frequent practice in Europe and the USA. The two major advantages of the use of iohexol are its stability at room temperature, −20 °C, and −80 °C [86,87], and the existence of an EQA (Equalis AB, Uppsala, Sweden) [88].

#### 2.4.1. Comparison between Clearance of Contrast Media and Inulin Clearance

There is only limited evidence that urinary iohexol clearance is a valid method to measure GFR [71,89,90], whereas the evidence for plasma clearance is at least moderately strong to consider it a valuable alternative to urinary inulin clearance [51,68,71,91,92,93,94]. Differences between plasma iohexol clearance and urinary inulin clearance are smaller than those observed with urinary iohexol clearance. No standardized protocol is currently available for determining mGFR based on plasma iohexol disappearance [95], and no study has appropriately compared the performance of single- and multiple-sample iohexol methods with urinary inulin clearance as the gold standard reference [34]. In a systematic review, the performance of plasma iothalamate clearance was substantially worse to determine GFR in comparison with the strong evidence for the validity of measurements based on urinary iothalamate clearance [71].

#### 2.4.2. Comparison between Clearances of Contrast Media

After a concurrent subcutaneous injection of both iothalamate (Conray™) and iohexol (Omnipaque™), urinary iohexol clearance was, on average, 15% lower than that of iothalamate across a wide range of GFRs. A decreased ultrafilterability of iohexol, perhaps due to higher protein binding, tubular iothalamate secretion, or tubular iohexol reabsorption, might explain this discrepancy [86].

#### 2.4.3. Limitations of the Use of Contrast Media

Iodinated contrast agents can trigger hypersensitivity or allergic reactions, can induce hyperthyroidism or thyroxtoxicosis, and can induce kidney failure at high doses. In comparison with imaging techniques, lower iohexol doses are used for GFR determinations with a lower risk of kidney failure. Iohexol is only registered as a contrast agent and not as a GFR marker. The off-label use of non-ionic contrast agents could lead to possible compensation claims by patients in case of adverse events [96].

### 2.5. Regulations for In Vitro Diagnostics

Both in the European Union (EU) and the United States (US), the policies issued by regulatory agencies regarding laboratory-defined test solutions prepared in vitro are becoming stricter. The US Food and Drug Administration (FDA) applies a comparable definition for diagnostic tools to the European Medicine Agency (EMA), specifying what is essential for safe and effective use [97,98]. In the EU, the EMA formulated a new set of in vitro diagnostic regulations (IVDR) which will become fully effective on 26 May 2022. This implies that diagnostic laboratories are experiencing difficulties in providing the required evidence and qualifications for their reagents, as the test procedures are becoming stricter. Under the current conditions, approximately 80% of in vitro diagnostics on the EU market are self-assessed for conformity by the manufacturer, and only a few have been evaluated by a notified body, contrary to what is stipulated by IVDR [99]. In the future, the use of radiodiagnostics would only be permitted if the manufacturer can provide evidence that it is compliant with the regulations and requirements of a diagnostic companion, not only for imaging applications but also for kidney function measurement.

## 3. A Potential Solution: A Multipanel Set of Markers

Given the known limitations of serum creatinine and exogenous biomarkers, and the widespread need for GFR estimations that are as precise as possible, a potential alternative approach is a combination of endogenous filtration markers in a panel from a single blood draw, downplaying the contribution of non-GFR determinants of each endogenous biomarker due to the large number of biomarkers and reducing the error in GFR estimation [100,101]. Potential non-GFR determinants of all endogenous filtration markers are the rate of generation by metabolic processes, the rates of kidney tubular secretion and reabsorption, and the rate of extrarenal elimination [101]. Short-term fluctuations in the true GFR affect serum concentrations of filtration markers more slowly than clearance values, and measurement of serum concentrations is less complex than clearance determinations. The ideal multipanel set of biomarkers should estimate GFR at least as accurately as mGFR, without the need for specification of demographic and clinical characteristics. More specifically, it would be preferable to avoid specification of race for GFR estimations [102], as well as of age and sex due to variations across different conditions, such as illness, diet, and geography. Standardization of GFR for body surface area may also not be optimal because of body composition variation [103].

Data integration with large-scale datasets, including DNA and RNA sequence data, metabolomics data, and proteomics data from individuals and groups of patients along the genotype–phenotype continuum of chronic kidney disease (CKD), might be helpful to develop a multimetabolite panel for GFR determination [104]. Metabolomics has the potential to be revolutionary in the field of GFR estimation by a rapid screening of thousands of metabolites which are often primarily cleared by glomerular filtration and could serve as alternative filtration markers if a strong correlation with mGFR can be demonstrated. Another major advantage of this technique might be the development of robust targeted mass spectrometry assays that are both accurate and easily included in multiplex panels [103]. Additionally, the utility of proteomics for predicting CKD incidence and prognosis has recently been demonstrated [105,106]. Computational approaches such as machine learning allow combining high-dimensional datasets in which the number of variables exceeds the number of clinical outcome observations [104]. A successful integration of data types across domains using similarity network fusion [107] and multi-omics factor analysis [108] can provide new insights, leading to better GFR estimation.

Although the optimal composition of such multimetabolite eGFR panels is still to be determined, low-molecular weight serum proteins and metabolic waste products have been identified as candidate filtration markers [109]. In the African American Study of Kidney Disease and Hypertension (AASK), and in the Multi-Ethnic Study of Atherosclerosis (MESA) [103], more than one quarter of the metabolites measured using non-targeted assays were significantly correlated with mGFR. A stronger correlation was observed between a dozen metabolites (Table 2) and mGFR in comparison with serum creatinine. Repeat testing in both cohorts using targeted assays for a subset of these promising metabolites resulted in an increased strength of correlation with mGFR. A more accurate estimation of mGFR was observed with targeted assays for panels of metabolites without creatinine than the CKD-EPI eGFRcr equation.

An acceptable, convenient, and widely available multimetabolite panel should be further validated in diverse study populations before clinical implementation can be considered. The eGFR panel might be as accurate as mGFR if novel statistical approaches are used that take into account the error of mGFR in estimating the true GFR [101]. The use of a mutimetabolite panel will increase the cost of GFR estimation, but if this improves accuracy and generalizability across populations and avoids specification of race, the benefit may be worth the cost [110].

## 4. Conclusions

GFR evaluation is crucial to clinical practice, public health, and research. In more detail, it is essential to explain signs, symptoms, and laboratory abnormalities that might be related to kidney disease, for drug development and dosing, and for detecting, treating, and estimating the prognosis of CKD, which is associated with increased morbidity and mortality [111].

Although widely used, the most accurate creatinine-based GFR-estimating equation for use in diverse populations (CKD-EPI 2009 creatinine equation) has its limitations. The main limitation of this equation is the imprecision in estimating mGFR, despite standardization of serum creatinine assays. This might be explained by variations in muscle mass and diet that are not adequately modeled by including age, sex, and ethnicity in the equation. Current creatinine-based equations have lower accuracy in ethnic groups other than white individuals and African Americans. In patients with comorbid conditions, substantially higher error rates can be observed [101]. Serum cystatin C has been proposed as an alternative marker as it is less influenced by muscle mass than serum creatinine, but its concentration is more affected by inflammation, adiposity, smoking, and levels of thyroid and corticosteroid hormones. The CKD-EPI 2012 cystatin C and creatinine-cystatin C equations were the first to be expressed for standardized serum cystatin C [109]. eGFR_cys_ and eGFR_cr_ have comparable accuracy, but a significantly higher accuracy is characteristic for eGFR_cr-cys_ in comparison with both single-marker equations owing to an improvement in precision rather than bias. Several cystatin C equations have been developed, which perform as well as, but not better than, the CKD-EPI 2012 equations. Although the 2012 CKD-EPI eGFR_cr-cys_ equation is more accurate than both the 2009 CKD-EPI eGFR_cr_ equation and the 2012 CKD-EPI eGFR_cys_ equation, the combined equation is not independent of eGFR_cr_ and requires specification of ethnicity. The 2012 CKD-EPI eGFR_cys_ equation can be used without specification of ethnicity but is not more accurate than the 2009 CKD-EPI eGFR_cr_ equation. The main limitation of these equations is that the non-GFR determinants of serum cystatin C are not fully understood. Despite improvement in bias compared with eGFR_cr_, substantial errors remain in eGFR_cys_ in chronically ill patients with heart and liver disease [101].

Besides the limitations of the use of serum creatinine and cystatin C, all mentioned mGFR procedures are complex and time-consuming and are associated with some degree of systematic (bias) or random (imprecision) error compared with the reference standard method (inulin clearance) [101]. The limited ^51^Cr-EDTA availability, the lack of certified reference materials for iohexol, the fact that use of iohexol as a kidney function assessment tool is an off-label application, and the lack of uniform and evidence-based GFR measurement protocols lead to varying mGFR results across studies and question the scientific and clinical merits of mGFR methods as the golden standard to evaluate kidney function. The smallest reported within-person CVs for repeated measurements on different days range from approximately 5 to 15%, with larger values for urinary clearance than for plasma clearance. This means that for a method without bias compared with the true GFR, a CV of 10% is equivalent to approximately 90% of measurements being within 15% of the true GFR. Sources of error compared with the reference method include the alternative clearance methods and non-ideal behaviors of the alternative exogenous filtration markers [19]. There is a call for standardization of mGFR at three levels: the marker to be used, the analytical method to be used, and a well-defined, uniform, and most optimal procedure to measure GFR [34]. On top of these technical arguments involved in these choices, regulatory issues are further constraining the clinical application of these methods, while they are also laborious, time-consuming, and costly. For these reasons, there is a call for the development of a panel of endogenous biomarkers which estimates GFR at least as accurately as mGFR, without the need for specification of demographic and clinical characteristics. However, some hurdles should be encountered before such a panel can be introduced. There is a lack of studies evaluating combinations of metabolites and low-molecular weight proteins in diverse populations to estimate kidney function. Nonetheless, the higher cost and the lack of widely available techniques to perform these measurements illustrate the gap between basic research and routine clinical practice of multimetabolite panels.

## Figures and Tables

**Table 1:** Proposed mGFR protocols according to the volume status and the anticipated level of eGFR [49].

Non-Edematous Patients	Plasma Iohexol mGFR Protocol			
	**Preferred Protocol**		**Equivalent Alternative Protocol**	
	**Initial Sample**	**Final Sample**	**Initial Sample**	**Final Sample**
eGFR > 60 mL/min/1.73 m^2^	2 h	4 h	2 h	5 h
eGFR 30–59 mL/min/1.73 m^2^	3 h	7 h	4 h 3 h 4 h	7 h 10 h 10 h
eGFR < 30 mL/min/1.73 m^2^	4 h	10 h	5 h	10 h
**Patients with significant edema**	**Urinary inulin clearance**			

eGFR, estimated glomerular filtration rate; mGFR, measured glomerular filtration rate.

**Table 2:** Overview of the correlations of several metabolites with mGFR [103].

Metabolite	Correlation with mGFR
X-11564 C-glycosyltryptophan pseudouridine erythronate N-acetylserine N-acetylthreonine 4-acetamidobutanoate N6-carbamoylthreonyladenosine myo-inositol urea N-acetyl-1-methylhistidine X-12411 creatinine	−0.744 −0.738 −0.723 −0.693 −0.622 −0.621 −0.594 −0.567 −0.542 −0.539 −0.513 −0.509 −0.504

mGFR, measured glomerular filtration rate.

## Data Availability

Not applicable.
